# Poor vector competence of the human flea, *Pulex irritans*, to transmit *Yersinia pestis*

**DOI:** 10.1186/s13071-021-04805-3

**Published:** 2021-06-10

**Authors:** Adélaïde Miarinjara, David M. Bland, James R. Belthoff, B. Joseph Hinnebusch

**Affiliations:** 1 Laboratory of Bacteriology, Rocky Mountain Laboratories, National Institute of Allergy and Infectious Diseases, NIH, Hamilton, MT USA; 2 Department of Biological Sciences, Boise State University, Boise, ID USA

**Keywords:** *Pulex irritans*, *Yersinia pestis*, Flea, Plague, Transmission

## Abstract

**Background:**

The human flea, *Pulex irritans*, is widespread globally and has a long association with humans, one of its principal hosts. Its role in plague transmission is still under discussion, although its high prevalence in plague-endemic regions and the presence of infected fleas of this species during plague outbreaks has led to proposals that it has been a significant vector in human-to-human transmission in some historical and present-day epidemiologic situations. However, based on a limited number of studies, *P. irritans* is considered to be a poor vector and receives very little attention from public health policymakers. In this study we examined the vector competence of *P. irritans* collected from foxes and owls in the western United States, using a standard protocol and artificial infection system.

**Methods:**

Wild-caught fleas were maintained in the laboratory and infected by allowing them to feed on human or rat blood containing 2 × 10^8^ to 1 × 10^9^ *Y. pestis*/ml. The fleas were then monitored periodically for infection rate and bacterial load, mortality, feeding rate, bacterial biofilm formation in the foregut (proventricular blockage), and ability to transmit *Y. pestis* after their single infectious blood meal.

**Results:**

*P. irritans* were susceptible to infection, with more than 30% maintaining high bacterial loads for up to 20 days. Transmission during this time was infrequent and inefficient, however. Consistent with previous studies, a low level of early-phase transmission (3 days after the infectious blood meal) was detected in some trials. Transmission at later time points was also sporadic, and the incidence of proventricular blockage, required for this mode of transmission, was low in fleas infected using rat blood and never occurred in fleas infected using human blood. The highest level of blockage and transmission was seen in fleas infected using rat blood and allowed to feed intermittently rather than daily, indicating that host blood and feeding frequency influence vector competence.

**Conclusions:**

Our results affirm the reputation of *P. irritans* as a feeble vector compared to rodent flea species examined similarly, and its vector competence may be lower when infected by feeding on bacteremic human blood.

**Graphic abstract:**

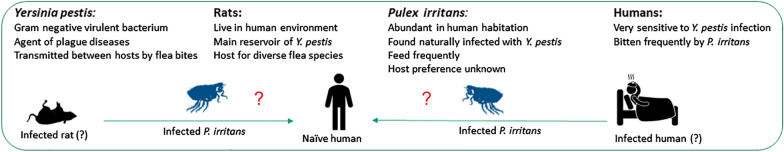

**Supplementary Information:**

The online version contains supplementary material available at 10.1186/s13071-021-04805-3.

## Background

Plague is principally a flea-borne disease of rodents, characterized by alternating periods of low transmission (enzootic plague) and high transmission (epizootic plague) within rodent populations, which vary in susceptibility to the disease [1]. Fleas become infected with the plague bacterium, *Yersinia pestis*, after taking a blood meal from an infected host with high bacteremia [2, 3]. Humans are most vulnerable when epizootic plague is decimating a susceptible peridomestic rodent population, and infected fleas from dead rodents seek new hosts [4].

Two modes of flea-borne transmission of *Y. pestis* have been described. Most prominently, transmission can occur after the bacteria form a cohesive biofilm in the proventriculus, a valve in the flea foregut that regulates blood flow between the esophagus and midgut during feeding. By impeding or completely blocking the flow of blood into the midgut, proventricular biofilm provokes persistent feeding attempts during which blood mixed with bacteria from the biofilm is regurgitated back into the bite sites [5–7]. A period of 5 days to a week or more is typically required for the buildup of sufficient biofilm to bring about this mode of transmission [8–10]. In a second mode of transmission, fleas can transmit within the first few days after having fed on a host with high-density bacteremia, when they next feed on a naïve host [8, 11, 12]. Originally referred to as mass transmission, because it rarely occurs unless several infected fleas feed simultaneously on a naïve host, it has been recently reexamined and termed early-phase transmission [13]. Early-phase transmission does not require the formation of a mature biofilm, but also occurs via regurgitation from a heavily colonized proventriculus [14–17].

Different flea species vary greatly in vector competence, the rate or efficiency at which they become infected and subsequently transmit *Y. pestis* after feeding on bacteremic blood. This variation is seen for both the early-phase [13, 18–22] and the proventricular blockage mechanisms [8, 9, 23]. For example, the rat flea *Xenopsylla cheopis,* the most studied, has high vector competence largely attributed to its susceptibility to develop proventricular blockage [9]. Early-phase transmission has been proposed as the driving force behind rapidly progressing epizootics vectored by fleas that do not block as readily [13, 22, 24]. However, vector competence analyses are quite limited for most flea species and are difficult to compare and sometimes conflicting because of the variety of experimental conditions used [9, 10]. For the same reasons, it is difficult to compare the relative efficiencies of the early-phase and proventricular biofilm modes for a given vector. The need for more systematic studies has been recognized [9, 15].

*Pulex irritans* presents an interesting case for human plague epidemiology. Although often referred to as the “human flea”, it actually parasitizes a wide range of domestic and wild animals worldwide, including birds, rodents, bats, large carnivores, wild ungulates, and domestic animals as well as humans, and its host distribution can vary according to geographical region [25–31]. Fleas such as *P. irritans* that can be abundant in houses and that bite humans have been suspected of vectoring human-to-human transmission of bubonic plague. This species has been found many times to be naturally infected during plague outbreaks and the prevalence of infection was greatest in high-risk plague areas [32–35]. Laboratory studies concerning the ability of *P. irritans* to transmit *Y. pestis*, using different approaches and experimental designs, have been reported since 1907. In some cases, presumably infected *P. irritans* were collected from houses of plague victims, or directly from a patient’s clothing, and transferred to a healthy guinea pig [32]. In other studies, uninfected *P. irritans* were allowed to feed on a plague-infected agonal rodent or human and then transferred to a naive guinea pig [8, 11, 12, 32]. With one exception [8] all of these studies examined early-phase transmission only, in which fleas were placed *en masse* on a naïve animal within a day or two after their infectious blood meal. This body of work demonstrated that *P. irritans* can acquire the infection during an infectious blood meal (from a human or rodent), can remain infected for at least 21 days, with virulent bacteria detected in feces for at least a few days after infection, and that infectious material deposited on the skin might infect animals through lesions from flea bites before the punctured skin could heal [12, 32]. However, when compared to an efficient vector such as *X. cheopis*, the vector competence of *P. irritans* has generally been considered insufficient for this species to play an important role during human plague epidemics (reviewed in [36]).

Nonetheless, investigators such as Blanc and Baltazard, who conducted the most documented works on *P. irritans* vectorial capacity, advanced their theory of inter-human plague transmission mediated by *P. irritans*. They claimed that the low vector competence of *P. irritans* is compensated for by its high abundance in the human environment and its potential for mass transmission. Their theory was supported by particular plague epidemiological settings in North Africa, the Middle East [32, 37–39], and other parts of the world where the classic rat flea to human transmission was difficult to prove, or seemed insufficient to explain the rapid spread of bubonic plague [40, 41]. However, in critical reviews, some authors concluded that the rare instances and experimental conditions in which the human flea transmitted the plague bacterium were not sufficient to support an important role for this flea in human-to-human transmission in natural conditions [42].

To bring more insight and resolution to the potential role of *P. irritans* in plague transmission, we conducted experimental infection and transmission assays using controlled, standardized protocols made possible by the use of an artificial feeding and infection system. Here we report the results and discuss their implications to plague epidemiology.

## Methods

### Flea collection and maintenance

From 2017 to 2020, adult *P. irritans* were periodically collected from western burrowing owls (*Athene cunicularia hypugaea*) in Idaho [43] and shipped live to the Rocky Mountain Laboratories. *Pulex irritans* were also collected from foxes (*Vulpes vulpes*) trapped locally and transported directly to the lab. Upon arrival, fleas were maintained at 21 °C, 75% relative humidity (RH) and usually infected within 1 day after receiving them, but for some experiments they were fed for 1 to 9 days on sterile defibrinated blood before infection (Additional file 1: Table S1).

### Flea infections

Fleas were infected by allowing them to feed on 5 ml of defibrinated rat (*Rattus norvegicus*) or human blood (both from BioIVT, NY, USA) containing 2 × 10^8^ to 1 × 10^9^ *Y. pestis* per ml, using an artificial feeding device system [44]. A *Y. pestis* KIM6 + strain containing the green-fluorescent-protein-expressing plasmid pAcGFP1 (Clontech/Takara Bio) was used for all experiments and was prepared and added to the blood meal as previously described [10]. Fleas were allowed to feed for 1 h through either a stretched Parafilm M membrane or mouse skin that was attached to the bottom of the artificial feeding device. After the feeding period, fleas were collected, immobilized by cold and CO_2_ exposure, and examined using a dissecting microscope. Only fleas displaying fresh red blood in the midgut were included in the experiments. A sample of these was immediately frozen at −80° C for later determination of the primary bacterial load in fleas, and the remainder maintained in capsules kept at 21 °C and 75% RH. A dilution series of the blood was plated and CFUs counted to obtain the bacterial concentration in the infectious blood meal. When the number of fleas received was sufficiently high, a portion of them was separately fed sterile blood on the same day to serve as matched uninfected controls.

### Transmission tests

Following infection, fleas were provided sterile maintenance blood meals every 1 to 3 days, using the same artificial feeding system and blood source. Mortality, feeding rate (percentage of fleas with fresh red blood meal in the midgut only), and evidence of proventricular blockage, either partial (red blood in esophagus and midgut) or complete (red blood in the esophagus only), were recorded. Transmission during these maintenance feedings was assessed as described previously [10]. Immediately after the feeding period, the blood was removed, and the interior of the feeding device was rinsed five times with 3 ml of phosphate buffered saline (PBS) to collect residual blood. The blood (in 200 µl portions) was spread onto blood agar plates containing 100 µg/ml carbenicillin; pooled washes were similarly plated after centrifugation and resuspension of the pellet in a small volume of PBS. Colony forming units (CFUs) were counted after 48 h incubation at 28 °C to determine the number of *Y. pestis* transmitted by the group of feeding fleas. At different time points after infection, a sample of fleas that had fed during the transmission test was placed at −80 °C. Flea samples collected at the different time points post-infection were later thawed, then surface-sterilized, and individually triturated and plated as previously described [45] to monitor the infection rate and the bacteria load per flea. The number of infected fleas that fed during a transmission test was estimated by multiplying the total number of fleas that fed by the infection rate determined on that day.

### Blood meal size measurement

To determine the average volume of blood consumed by *P. irritans* in a blood meal, a sample of 17 uninfected female fleas (collected from a fox) were starved for 2 days and then immobilized and weighed as a group using a microbalance (Sartorius AG, Germany) before allowing them to feed on sterile defibrinated dog blood through a mouse skin membrane attached to an artificial feeding device. After a 1 h feeding period, those fleas that had taken a blood meal were collected and reweighed. The average weight increase (0.34 mg; the difference in the average post-feed and pre-feed weights) was divided by 1.06, an average specific gravity of blood, to give the average blood meal volume (0.32 µl; Additional file 1: Table S2).

### Statistics

Differences in mortality rate between infected and uninfected fleas were analyzed by log-rank test, and differences in flea bacterial loads analyzed by *t*-test or ANOVA, using GraphPad Prism software.

## Results

Fleas collected from burrowing owls and foxes were verified as *Pulex irritans* by standard classification keys based on morphology [46]. In addition, sequence analysis of PCR-amplified segments of the ITS2 region and the COII and 16S genes [47] were identical to *P. irritans* sequences (Genbank accession numbers KX98286, KY073316, and GQ387497).

Fleas were infected and maintained using four experimental conditions in this study to assess the effects of different characteristics of mammalian blood and the frequency of blood-feeding, both of which can significantly influence flea vector competence and transmission dynamics [16, 45, 48]. Accordingly, fleas were infected using either human or rat blood and allowed to feed on sterile blood of the same type either daily or once every 2 to 3 days (Additional file 1: Table S1). In all cases, the infectious blood meal on day 0 contained > 10^8^ *Y. pestis*/ml, the high bacteremia level required to potentiate early-phase transmission [49]. When the number of available fleas was sufficient, a portion was segregated and fed only on sterile blood to serve as an uninfected control group.

### Group I: Fleas infected using human blood and fed sterile human blood daily starting from day 1–2 after infection

**Fig. 1:**
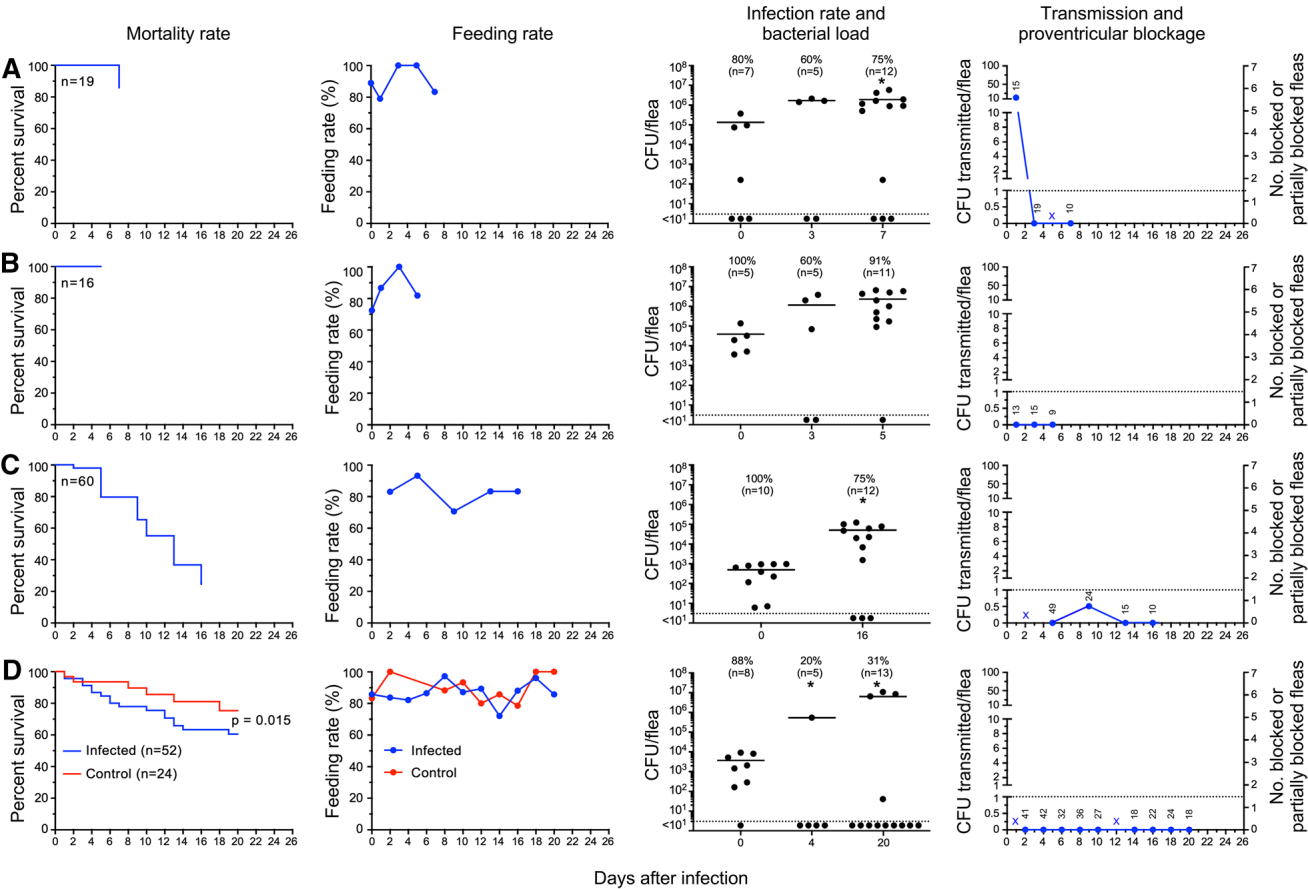
*P. irritans* fleas infected using human blood and fed sterile human blood daily from day 1–2 to day 20 after infection. Survival rates, feeding rates (percentage of fleas that fed on a given day), infection rates, bacterial load per infected flea, transmission and proventricular blockage dynamics are shown for four independent experiments (**A**–**D**). **A** and **B** were short-term experiments in which only early-phase transmission was assessed. The starting number of infected fleas (*n*) is indicated on the survival graphs. The infection rates (%) at different times after infection and the number of individual fleas assayed (*n*) is indicated on the infection rate and bacterial load graphs, with the horizontal line marking the mean CFU per infected flea and the dashed line the level of detection for the CFU plate count assay. *, bacterial load per flea significantly greater than the day 0 count (*p* < 0.05). Transmission tests were done on the days indicated; the numbers on the transmission graphs are the number of infected fleas that fed on the test blood before it was plated, and the dashed line marks the level of 1 CFU recovered per infected flea bite. X indicates that fleas were fed but scheduled transmission test was not done due to disruption of the feeding membrane during blood collection. No evidence of partial or complete proventricular blockage was detected in any of the fleas in this group of experiments

Because *P. irritans* reportedly take frequent blood meals [26], this group would presumably be most relevant to the human-to-human transmission scenario. Two of these experiments involved small numbers of fleas, and only early-phase transmission (the first feeding opportunity after infection) was evaluated (Fig. 1A, B). Infection rates varied, even in flea samples that were collected after the 1-h feeding period. This is likely due to the fact that *P. irritans*, as is typical of fleas, take small blood meals (on average, only 0.32 µl for females; Additional file 1: Table S2), and appear to excrete part of it during or soon after feeding, as evidenced by the presence of fresh fecal spots in the feeding capsule. In addition, even within an hour of feeding, the blood meal in the midgut was typically dark instead of bright red, with some already present in the hindgut, indicating that *P. irritans* digest a blood meal rapidly. Nevertheless, the majority of fleas (75 to 91%) were still infected with large numbers of *Y. pestis* 5 to 7 days after their infectious blood meal, when these experiments were terminated (Fig. 1A, B). Early-phase transmission, 1 to 2 days after the infectious blood meal, was detected in only one of the two experiments.

In two other experiments, fleas were maintained and monitored for 2 to 3 weeks (Fig. 1C, D). The average number of *Y. pestis* per infected flea increased at least tenfold during the course of the experiments, indicating that *Y. pestis* can replicate and stably colonize this flea species, but again the infection rates varied, with 31 to 75% of fleas still infected at the end of these experiments (Fig. 1 C, D). There was no evidence of early-phase transmission in the one experiment in this subgroup in which it was assessed (Fig. 1D), but transmission was detected on day 9 post-infection (Fig. 1C). No transmission was ever detected in the other experiment in trials between days 5 and 16 after infection (Fig. 1D).

In addition to infection rate, bacterial load per flea, and transmission, we also monitored feeding rate, survival, and any development of proventricular blockage. Daily feeding rates ranged from 70 to 100%, and no evidence of partial or complete blockage was ever detected. Survival rates in the two longer-term experiments were quite different: in one case (Fig. 1C), 75% of the fleas had died by day 16; in the other, only 40% by day 20 (Fig. 1D). In the latter experiment, uninfected control fleas had lower mortality (25%) than the infected cohort (40%), but this difference was not statistically significant. Data for the group I experiments are summarized in Table 1.

**Table 1:** Transmission by *Pulex irritans* infected using human blood and fed sterile human blood daily starting from day 1–2 after infection (group I)

Day post-infection	Total fleas	Fed flea number	Infection rate (%)	Mean CFU/flea	Blocked (B) fleas	Partially blocked (PB) fleas	B + PB fleas (%)	Total CFU transmitted	CFU transmitted per fed flea	
Exp#1 (owl; Fig. 1A)										
0	27	19	80	1.3 × 10^5^	–	–	–	–	–	
1	19	15	ND	ND	0	0	0	188	12.5	
3	19	19	60	1.7 × 10^6^	0	0	0	0	0	
5	14	14	ND	ND	0	0	0	ND	ND	
7	12	10	75	1.9 × 10^6^	0	0	0	0	0	
Exp #2 (owl; Fig. 1B)										
0	29	16	100	3.9 × 10^4^	–	–	–	–	–	
1	15	13	ND	ND	0	0	0	0	0	
3	15	15	60	1.9 × 10^6^	0	0	0	0	0	
5	11	9	91	2.5 × 10^6^	0	0	0	0	0	
Exp #3 (fox; Fig. 1C)										
0	ND	60	100	5.5 × 10^4^	–	–	–	–	–	
2	59	49	ND	ND	0	0	0	ND	ND	
5	43	40	ND	ND	0	0	0	0	0	
9	34	24	ND	ND	0	0	0	13	0.5	
13	18	15	ND	ND	0	0	0	0	0	
16	12	10	75	4.6 × 10^6^	0	0	0	0	0	
Exp #4 (fox; Fig. 1D)										
0	70	52	88	1.8 × 10^6^	–	–	–	–	–	
2	49	41	ND	ND	0	0	0	0	0	
4	47	42	20	1.8 × 10^6^	0	0	0	0	0	
6	37	32	ND	ND	0	0	0	0	0	
8	37	36	ND	ND	0	0	0	0	0	
10	31	27	ND	ND	0	0	0	0	0	
12	28	25	ND	ND	0	0	0	ND	ND	
14	25	18	ND	ND	0	0	0	0	0	
16	25	22	ND	ND	0	0	0	0	0	
18	25	24	ND	ND	0	0	0	0	0	
20	21	18	23	2.0 × 10^6^	0	0	0	0	0	

*ND* not determined. The source of the fleas used for the experiments (owl or fox) is indicated

### Group II: Fleas infected using human blood and subsequently fed sterile human blood every 2 days

**Fig. 2:**
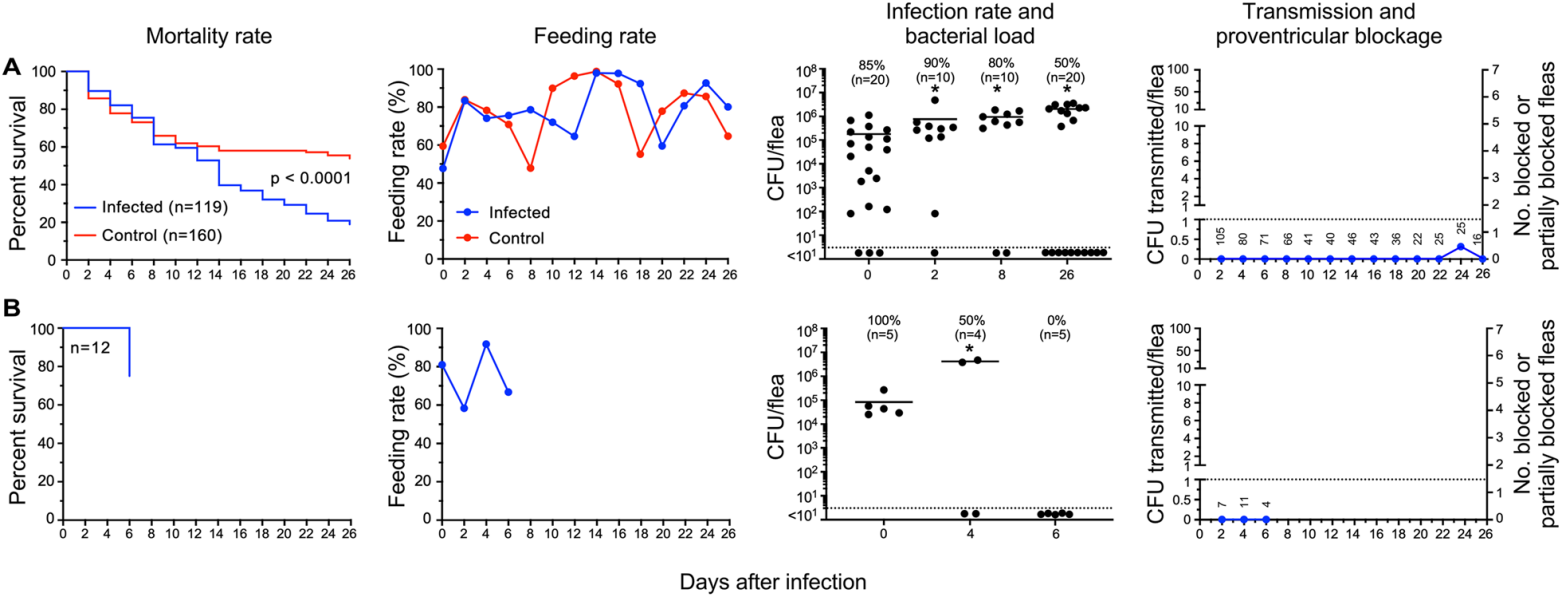
*P. irritans* infected using human blood and fed sterile human blood every 2 days. The results of two independent experiments (**A**, **B**) are shown; **B** was a short-term experiment in which only early-phase transmission was assessed. See Fig. 1 legend for details. No evidence of partial or complete proventricular blockage was detected in any of the fleas

Two experiments were conducted in which fleas were fed every other day instead of daily after an infectious human blood meal. In the first (Fig. 2A), 80% of the fleas retained the infection after 8 days, and 50% were still infected at the end of the experiment on day 26 post-infection, with an approximately tenfold increase in average *Y. pestis* per flea compared to day 0. Mortality of the infected fleas (80% by 26 days) was significantly higher (*p* < 0.001) than mortality of the uninfected control fleas (46%). This suggests that *Y. pestis* infection caused considerable morbidity, even though no evidence of partial or complete proventricular blockage was ever detected in any flea. *P. irritans* has been described as a frequent feeder but feeding rates in this group were no higher than for fleas that were allowed to feed daily. No early-phase transmission was detected upon the first blood meal after infection on day 2, even though approximately 95 infected fleas fed. No transmission occurred at all except on day 24, when eight CFU were recovered from the reservoir of blood fed upon by 25 fleas, none of which appeared to be blocked or partially blocked. The second experiment (Fig. 2B) involved small numbers of fleas and only proceeded through the early phase, and no transmission was detected. Data are summarized in Table 2.

**Table 2:** Transmission by *Pulex irritans* infected using human blood and subsequently fed sterile human blood every 2 days (group II)

Day post-infection	Total fleas	Fed flea number	Infection rate (%)	Mean CFU/flea	Blocked (B) fleas	Partially blocked (PB) fleas	B + PB fleas (%)	Total CFU transmitted	CFU transmitted per fed flea
Exp#1 (fox; Fig. 2A)									
0	292	119	85	1.8 × 10^5^	–	–	–	–	–
2	126	105	90	7.6 × 10^5^	0	0	0	0	0
4	108	80	ND	ND	0	0	0	0	0
6	94	71	ND	ND	0	0	0	0	0
8	84	66	80	9.5 × 10^5^	0	0	0	0	0
10	57	41	ND	ND	0	0	0	0	0
12	62	40	ND	ND	0	0	0	0	0
14	47	46	ND	ND	0	0	0	0	0
16	44	43	ND	ND	0	0	0	0	0
18	39	36	ND	ND	0	0	0	0	0
20	37	22	ND	ND	0	0	0	0	0
22	31	25	ND	ND	0	0	0	0	0
24	27	25	ND	ND	0	0	0	8	0.3
26	20	16	50	2.0 × 10^6^	0	0	0	0	0
Exp #2 (owl; Fig. 2B)									
0	21	12	100	1.4 × 10^6^	–	–	–	–	–
2	12	7	ND	ND	0	0	0	0	0
4	12	11	50	1.7 × 10^6^	0	0	0	0	0
6	6	4	0	0	0	0	0	0	0

*ND* not determined. The source of the fleas used for the experiments (owl or fox) is indicated

### Group III: Fleas infected using rat blood and fed sterile rat blood daily starting from day 2 after infection

**Fig. 3:**
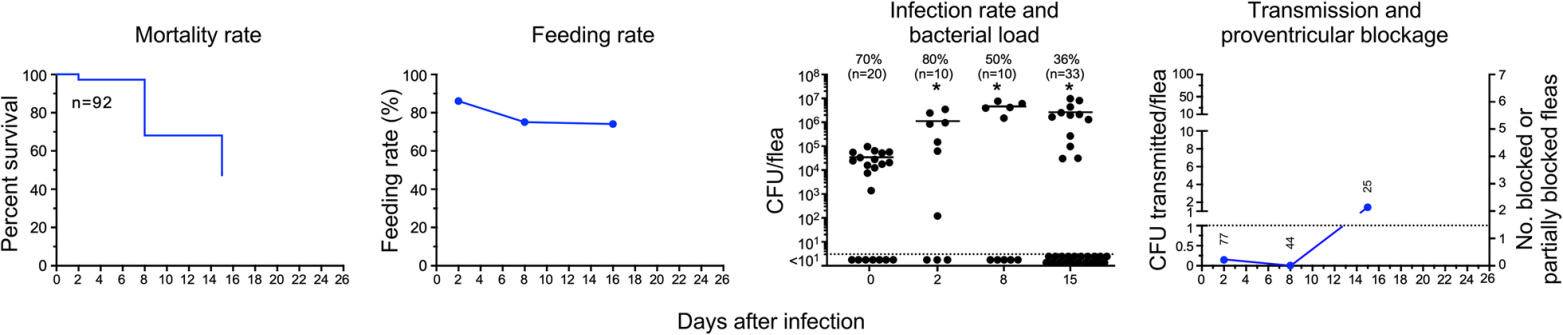
*P. irritans* fleas infected using rat blood and fed sterile rat blood daily from day 2 after infection; see Fig. 1 legend for details. No evidence of partial or complete proventricular blockage was detected in any of the fleas in this experiment

Although not their preferred hosts, *P. irritans* will feed on small rodents, and it has been proposed that after feeding on a bacteremic rodent, this flea could later transmit *Y. pestis* to other hosts [12]. After *P. irritans* fed on highly bacteremic rat blood, the infection rate was comparable to that of fleas infected using human blood (Fig. 3). In this experiment in which fleas infected using rat blood were fed daily thereafter with sterile rat blood, 36% were still infected 15 days later, with high bacterial loads per flea. As with fleas infected using human blood, infected flea mortality was high (53% at day 15). Early-phase transmission was detected, with 12 CFU transmitted by the 77 fleas that fed on day 2. Later transmission occurred on day 15 after infection, when 41 CFU were recovered from the blood fed upon by 25 fleas, although these fleas showed no obvious signs of partial or complete blockage (Fig. 3). Data for the group III experiment is summarized in Table 3.

**Table 3:** Transmission by *Pulex irritans* infected using rat blood and fed sterile rat blood daily starting from day 2 after infection (group III)

Day post-infection	Total fleas	Fed flea number	Infection rate (%)	Mean CFU/flea	Blocked (B) fleas	Partially blocked (PB) fleas	B + PB fleas (%)	Total CFU transmitted	CFU transmitted per fed flea
Exp #1 (fox; Fig. 3)									
0	ND	92	70	3.4 × 10^4^	–	–	–	–	–
2	90	77	80	1.1 × 10^6^	0	0	0	12	0.15
8	59	44	50	4.6 × 10^6^	0	0	0	0	0
15	34	25	36	2.6 × 10^6^	0	0	0	41	1.6

*ND* not determined. The source of the fleas used for the experiments (owl or fox) is indicated

### Group IV: Fleas infected using rat blood and subsequently fed sterile rat blood every 2 to 3 days

**Fig. 4:**
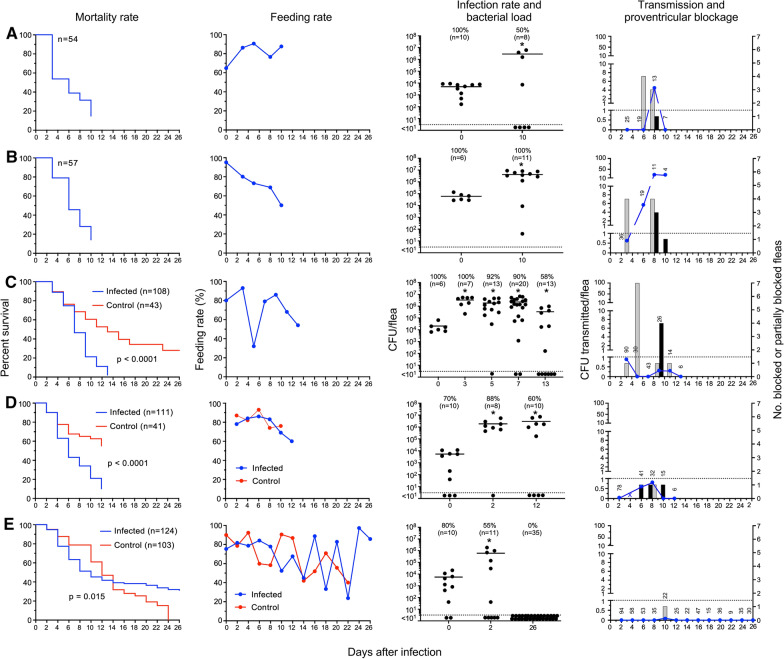
*P. irritans* fleas infected using rat blood and fed sterile rat blood every 2 to 3 days. The results of five independent experiments are shown in (**A**–**E**); see Fig. 1 legend for details. Grey bars indicate the number of partially blocked fleas and black bars the number of completely blocked fleas diagnosed immediately after feeding

Mortality of infected fleas in this group was again high, with most of the fleas dead by 10 to 13 days after infection, except in one experiment in which all surviving fleas had cleared themselves of infection (Fig. 4). Mortality of uninfected control fleas was also high within only 3 weeks, and the mortality of infected fleas was even higher. Feeding rates were variable and somewhat lower than for other groups, even though the fleas had the opportunity to feed only every other day, which may account in part for the high mortality. Infection rates and bacterial levels in infected fleas were similar to those of the other three experimental groups.

**Fig. 5:**
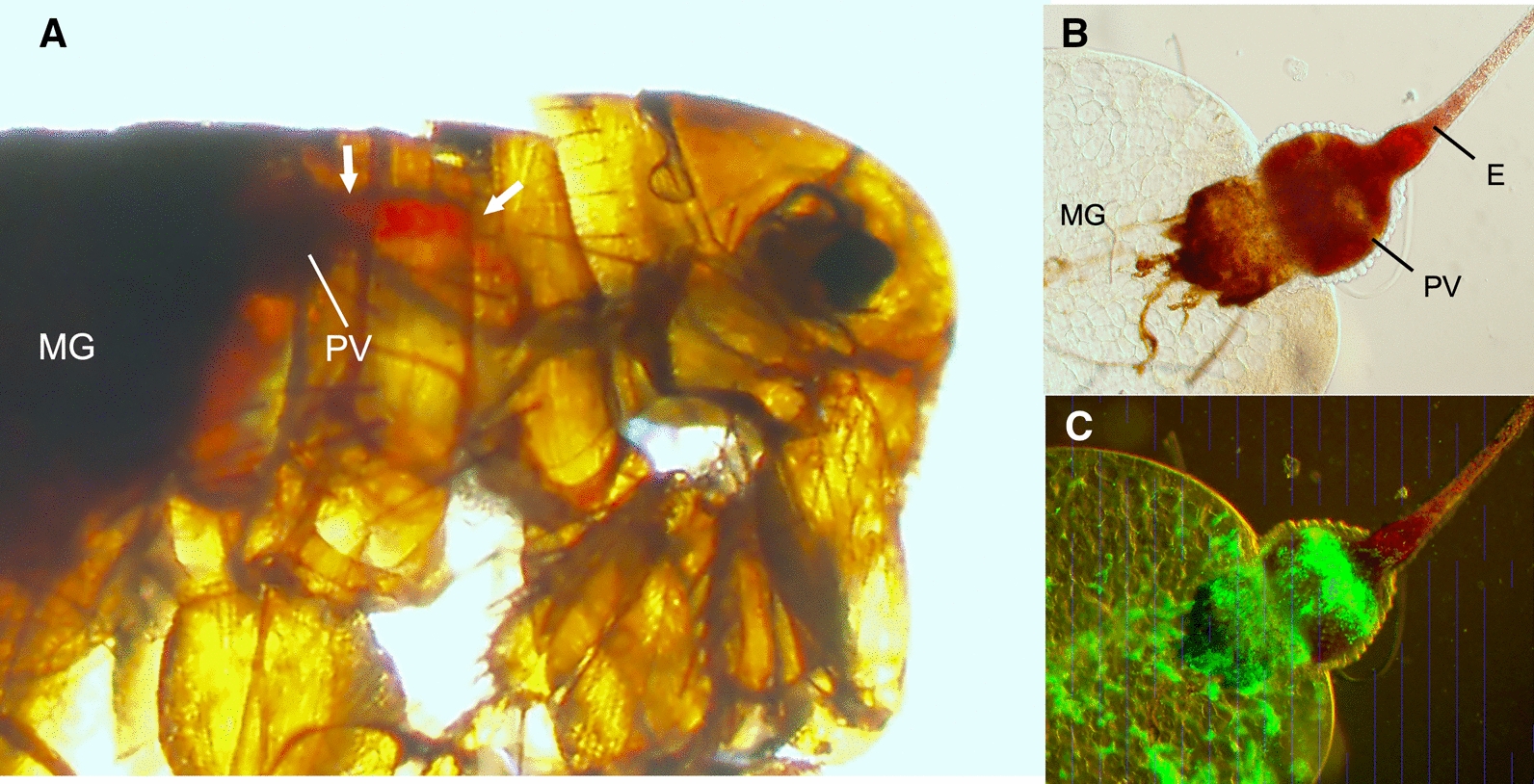
Proventricular blockage in *P. irritans* infected with green fluorescent protein-expressing *Y. pestis* in rat blood. **A** Immediately after this flea attempted to feed 6 days after infection, fresh red blood was present only in the esophagus (delineated by the white arrows) anterior to the proventriculus (PV). No fresh blood was present in the midgut (MG), which contains only dark-colored remnants of previous blood meals. Light (**B**) and fluorescence (**C**) microscopy of the digestive tract dissected from this flea shows the dense bacterial biofilm aggregate that fills the proventriculus and blocked the flow of blood from the esophagus (**E**)

Most notably, in contrast to the previous three experimental groups, partially and/or completely blocked fleas were observed in each of the five experiments in this group (Fig. 4). Twelve fleas showed the classic blocked picture of fresh blood only in the esophagus after attempting to feed, with none in the midgut (Fig. 5), and 27 appeared to be partially blocked. Complete blockage was not observed in one experiment in which a majority of fleas eliminated the infection and feeding rates varied widely, but one flea in this experiment developed partial blockage (Fig. 4E). Early-phase transmission was seen in two of the five experiments, both of which were associated with the appearance of partial blockage, 3 days after infection. In keeping with the incidence of blockage, later-phase transmission efficiency was much higher in this group and in four instances resulted in higher numbers of CFU transmitted per flea, for example on day 8 in one experiment (Fig. 4B), in which 7 of the 11 fleas that fed were either blocked or partially blocked and transmitted an average of 28 CFU per flea. Cumulatively, 12 of 454 fleas (2.6%) in this group that fed in the transmission trials were completely blocked, and 27 (5.9%) were partially blocked. Data for the group IV experiments are summarized in Table 4.

**Table 4:** Transmission by *Pulex irritans* using rat blood and subsequently fed sterile rat blood every 2 to 3 days (group IV)

Day post-infection	Total fleas	Fed flea number	Infection rate (%)	Mean CFU/flea	Blocked (B) fleas	Partially blocked (PB) fleas	B + PB fleas (%)	Total CFU transmitted	CFU transmitted per fed flea
Exp #1 (owl; Fig. 4A)									
0	ND	54	100	4.9 × 10^3^	–	–	–	–	–
3	29	25	ND	ND	0	0	0	0	0
6	21	19	ND	ND	0	4	21	0	0
8	17	13	ND	ND	1	3	31	59	4.5
10	8	7	50	2.9 × 10^6^	0	0	0	0	0
Exp #2 (owl; Fig. 4B)									
0	ND	57	100	6.1 × 10^4^	–	–	–	–	–
3	36	36	ND	ND	0	4	11	23	0.6
6	19	19	ND	ND	0	0	0	107	5.6
8	11	11	89	ND	3	4	64	306	28
10	4	4	100	4.2 × 10^6^	0	1	25	97	24
Exp #3 (fox; Fig. 4C)									
0	ND	108	100	2.1 × 10^4^	–	–	–	–	–
3	97	90	100	3.3 × 10^6^	0	1	1	81	0.9
5	94	30	92	1.9 × 10^6^	0	7	23	0	0
7	55	43	90	2.3 × 10^6^	0	0	0	0	0
9	30	26	ND	ND	4	1	19	7	0.3
11	20	14	ND	ND	1	0	7	4	0.3
13	11	6	58	3.4 × 10^5^	0	0	0	0	0
Exp #4 (fox; Fig. 4D)									
0	ND	111	100	5.3 × 10^3^	–	–	–	–	–
2	101	78	88	1.9 × 10^6^	0	0	0	3	0.04
4	73	61	ND	ND	0	0	0	ND	ND
6	48	41	ND	ND	1	0	2	25	0.6
8	39	32	ND	ND	1	1	3	27	0.8
10	22	15	ND	ND	1	0	7	0	0
12	10	6	58	2.9 × 10^6^	0	0	0	0	0
Exp #5 (fox; Fig. 4E)									
0	ND	124	88	3.7 × 10^3^	–	–	–	–	–
2	117	94	ND	ND	0	0	0	0	0
4	74	58	20	5.4 × 10^5^	0	0	0	0	0
6	63	53	ND	ND	0	0	0	0	0
8	45	35	ND	ND	0	0	0	0	0
10	42	22	ND	ND	0	1	5	2	0.1
12	37	25	ND	ND	0	0	0	0	0
14	49	22	ND	ND	0	0	0	0	0
16	53	47	ND	ND	0	0	0	0	0
18	45	15	ND	ND	0	0	0	0	0
20	44	36	ND	ND	0	0	0	0	0
22	38	9	ND	ND	0	0	0	0	0
24	36	35	ND	ND	0	0	0	0	0
26	35	30	0	0	0	0	0	0	0

*ND* not determined. The source of the fleas used for the experiments (owl or fox) is indicated

## Discussion

Vector competence is the ability of individuals in a vector population to become infected and transmit a given strain of pathogen [50]. Although many different flea species are involved in enzootic plague cycles throughout the world, their vector competence varies [1, 8, 9, 23]. The volume of the blood meal, the frequency of feeding and defecation, and the characteristics of host blood and digestion can influence the rates at which fleas clear themselves of *Y. pestis* or develop chronic infection [16, 45, 48]. Subsequent regurgitative transmission efficiency can be influenced by host blood characteristics and digestion, the propensity to develop proventricular blockage, and the anatomical structure and musculature of the proventricular valve [10, 15, 16, 45]. *Pulex irritans* is considered to be a poor vector, and in this study, we systematically reevaluated both aspects of its vector competence: the infection rate and the bacteria load per flea at different time points after an infectious blood meal were monitored. We also investigated the transmission efficiency during both the early-phase and later time period after an infectious blood meal, and the ability of *P. irritans* to develop proventricular blockage. Furthermore, we compared the effect of human or rat blood source and feeding frequency on *Y. pestis* infection and transmission dynamics.

We found that after feeding on either human or rat blood with a high bacteremia level (> 10^8^ *Y. pestis*/ml), *P. irritans* could maintain infection up to 26 days, but with a variable infection rate ranging from 0 to 100% (median = 54%) during this period. This result is in accordance with previous studies, in which naïve laboratory animals developed disease when inoculated with triturates of pooled *P. irritans,* or their feces, up to 20 days post-infection [8, 32, 51, 52]. The *P. irritans* infection rate was low when compared to comparable studies with efficient rodent flea vectors such as *X. cheopis* [10]. However, those fleas that were stably infected had high bacterial loads, comparable to those achieved in *X. cheopis*. The average bacterial burdens increased significantly during the first week after infection, indicating that *Y. pestis* is able to grow in the *P. irritans* gut.

We evaluated the transmission potential of infected *P. irritans* during the early phase, i.e., less than 5 days after the infectious blood meal. Early-phase transmission was detected only once in the four trials with fleas fed on infected human blood (7 to 105 fleas feeding per trial), and four times in the six trials with fleas infected using rat blood (25 to 94 fleas feeding per trial). Both the frequency of transmission and the CFU number transmitted per flea were low compared to what was reported for early-phase transmission by *X. cheopis* and *Oropsylla montana* fleas infected using rat blood and maintained in the same conditions [16, 18, 49].

We further monitored proventricular blockage and transmission beyond the early phase, which had only been examined once before for *P. irritans* [8]. Strikingly, proventricular blockage was only observed in fleas infected using rat blood, and never in fleas infected using human blood. Blockage developed within the first 2 weeks after infection, and in one transmission trial 8 days after infection, 7 of the 11 fleas that fed were either partially or completely blocked. Partially blocked *P. irritans* were observed as early as 3 days after infection, indicating that early-phase and biofilm-dependent transmission might overlap temporally. *Pulex irritans* were more likely to develop proventricular blockage when fed intermittently, rather than daily. Overall, approximately 2.6% of the fleas infected using rat blood that fed intermittently in the post-early-phase transmission trials were blocked. This blockage rate is much lower than observed for *X. cheopis* (~ 40%) and *O. montana* (~ 20%) under similar laboratory conditions [10]. Furthermore, blockage was detected only in fleas fed every 2–3 days after an infectious rat blood meal and not in fleas fed daily.

Attributes of the infecting host’s blood and the frequency of post-infection blood meals have previously been shown to influence vector competence. For example, the normal frequent (daily) feeding and excretion behavior of cat fleas (*Ctenocephalides felis*) leads to rapid clearance of *Y. pestis* from the digestive tract, but a high chronic infection rate and the development of proventricular blockage occur if the fleas feed only twice weekly [45]. Infection rates and bacterial burdens are higher in *X. cheopis* and *O. montana* infected using rat blood compared to mouse or rabbit blood [48], and hemoglobin solubility and digestion rate differences of mammalian blood in the flea gut have been correlated with subsequent transmission dynamics [16]. Notably, *X. cheopis* and *O. montana* infected using rat or guinea pig blood reflux a mixture of partially digested blood, hemoglobin crystals, and *Y. pestis* into the foregut soon after an infectious blood meal, and this post-infection esophageal reflux (PIER) phenomenon is associated with greater early-phase transmission efficiency [16]. Fleas infected using mouse or gerbil blood, which are digested more rapidly and whose hemoglobin is more soluble in the flea gut do not develop PIER, and early-phase transmission is lower. In this study, we noted that *P. irritans* digests its blood meals and excretes portions of it rapidly, and we did not observe PIER with either rat or human blood, although the partial blockage diagnosed in one rat-blood-infected flea on day 3 may have been due to PIER rather than to biofilm-dependent blockage. The dark, highly sclerotized exoskeleton of *P. irritans* makes PIER more difficult to detect in this flea than in others.

As with early-phase transmission, transmission by *P. irritans* during later times after infection was inefficient and sporadic. Transmission by fleas infected using human blood occurred in only 2 of 19 post-early-phase trials, with on average < 1 CFU transmitted per flea. In contrast, fleas infected using rat blood transmitted in 10 of 28 trials during the same time frame, with on average > 1 CFU transmitted in 6 trials and > 10 CFU in two of these. Thus, the ability to produce partial and complete blockage correlated with the greater transmission efficiency of *P. irritans* infected using rat vs. human blood. However, as has been noted before, feeding by blocked or partially blocked fleas did not always result in transmission; and conversely, transmission was sometimes observed when no blockage was detected among the fleas that fed. In the sole previous study of blockage rate and associated biofilm-dependent transmission, Burroughs (1947) reported that only 1 of 57 *P. irritans* became blocked, 11 days after an infectious blood meal from a moribund mouse. These 57 fleas were fed individually on guinea pigs daily (421 individual feedings over 7 weeks), and no transmissions were recorded [8].

After feeding on very highly bacteremic blood, a variable but significant percentage of fleas developed chronic infection with large numbers of *Y. pestis*, so the low transmission efficiency cannot be ascribed to an inability to infect the digestive tract of *P. irritans*. However, the infection rate associated with lower bacteremia levels was not assessed and would be an important consideration [49]. In our experiments, even though the infection rate was high during the early-phase, transmission efficiency was low. These results conform with previous studies, all of which reported successful transmission only when using numerous *P. irritans* to challenge test animals, the minimum number apparently being five fleas [12]. For example, in one study only 3 of 38 guinea pigs challenged with groups of ~ 20 *P. irritans* that had last fed on bacteremic rats developed plague [11]. In another study, groups of 10–15 *P. irritans* infected similarly were used to challenge 13 rats, two of which became sick [12]. Burroughs [8], using larger challenge groups of 60 and 80 *P. irritans* infected the previous day by feeding on a bacteremic mouse, reported successful transmission to two guinea pigs [8]. Interestingly, in 8 experiments in which 20–240 *P. irritans* were collected from humans who had recently succumbed to plague (or from the houses of recently deceased plague victims) and applied to a naïve guinea pig a day later, no transmissions resulted. Successful transmission only occurred in a 9th experiment in which 720 fleas recovered from six human plague victims were used to challenge a guinea pig [32]. It is not known how many of these fleas were infected and fed, but the low transmission efficiency of fleas from this natural setting may reflect lower terminal bacteremia levels in humans than in mice and rats [11, 53].

The advantages of the artificial feeding system used in this study are that the bacteremia level of the infectious blood meal can be controlled, the infection rate of the fleas used in transmission trials is monitored, and the number of CFUs transmitted into the blood reservoir from a known number of feeding fleas can be determined. In previous trials using laboratory animals, the initial infectious dose acquired by the fleas, as well as the subsequent feeding rates and infection status of the fleas used in transmission challenges were unknown. However, as also noted in all the previous studies using rodents, we found that *P. irritans* proved to be a difficult flea to maintain using the same laboratory conditions and experimental systems that have been used successfully with the rat flea *X. cheopis*, the North American ground squirrel flea *O. montana*, and the cat flea *C. felis* [10, 45]. Many experiments were fraught with high background mortality, which included even uninfected control fleas, even though *P. irritans* has been documented to live for months [26, 54]. The early-phase transmission experiments were least affected by this, and our results are consistent with the previously cited reports that *P. irritans* is an inefficient early-phase vector. In our experience, high background mortality can lead to inconsistent results regarding proventricular blockage-dependent transmission [2, 10]. This may account for the variation we observed among experiments within the groups. Due to the high mortality, the number of fleas available for transmission trials after 1 week was also often rather low. The reluctance of field-collected *P. irritans* to feed on laboratory animals has been proposed to explain the low survival [32]. In our artificial feeding system, we also observed that feeding rates were often not 100%, as would be expected for a flea that is supposed to take frequent blood meals. Another problem we occasionally encountered was leakage of the parafilm feeding membrane, precluding the inclusion of some transmission time-point data. Indeed, it is possible that some of the transmission we detected was not regurgitative but resulted from contamination of the blood reservoir from infected fecal deposits on the outer surface of the membrane. This may have resulted in some overestimation of transmission; however, transmission by fleas probing through infected feces on the skin has been proposed as an alternate means of transmission [11, 52]. This may be particularly pertinent for a flea like *P. irritans* that feeds and excretes frequently [26].

Another potential limitation of this study was the use of *P. irritans* strains collected from foxes and owls, which are principal hosts of this species in the western United States. *Pulex irritans* from North America have not been implicated in sylvatic plague transmission cycles, since their avian and carnivorous hosts are not susceptible to plague infection and they are rarely found to parasitize susceptible rodent species [43, 54]. A considerable degree of genomic divergence exists among different host-adapted *P. irritans* populations from different geographic areas, even though their anatomical morphology is identical [28]. It is not uncommon within pathogen vectors for different strains of the same species to have variable vector competence [50], and the importance of assessing the response of different flea strains of the same species to *Y. pestis* infection has been noted [8]. Thus, to get a more complete insight into *P. irritans* vectorial capacity, vector competence experiments with different strains of *P*. *irritans* (most importantly, human-adapted strains) will be necessary.

Despite being acknowledged as an inefficient vector, transmission upon the first feeding event after an infectious blood meal (early-phase or mass transmission) has been advanced as playing an important role in the vector potential of *P. irritans* to humans [13, 32, 55, 56]. In this scenario, the poor vector competence of *P. irritans* is compensated for by the large numbers of this flea that parasitize humans in some conditions. One possibility is that *P. irritans* infected by feeding on a peridomestic rodent dying of plague could subsequently transmit *Y. pestis* to a human. For example, in the Andean plague focus, guinea pigs are frequently maintained in human dwellings and are subject to plague [57, 58], and guinea pig blood induces early-phase transmission-enhancing PIER [16]. In support of the rodent-*P. irritans*-human scenario, we found that when infected using rat blood *P. irritans* could develop partial and complete blockage, which was associated with increased transmissibility. However, the so-called human flea associated with human dwellings in plague-endemic areas appears to have a high host preference for humans. In Madagascar, *P. irritans* has rarely been recovered from rodents captured inside houses, although it is the most abundant flea species sampled with light traps [35, 59]. Blood meal analyses conducted in Uganda and the Democratic Republic of Congo also revealed that *P. irritans* preferred human hosts [60].

Direct human-to-human transmission via *P. irritans* has also been proposed as a significant factor in plague outbreaks, including the second pandemic [32, 40, 41]. Our results with *P. irritans* infected by feeding on highly bacteremic human blood (> 10^8^ *Y. pestis*/ml) bear on this scenario. These fleas never developed even partial blockage, and transmission during both the early phase and later stages was the exception rather than a usual event, suggesting that the flea burden would have to be exceptionally high. This is supported by the failure of Blanc & Baltazard (1945) to transmit plague to a guinea pig unless it was challenged by 720 *P. irritans* collected from human plague victims [32]. Nevertheless, based on human plague outbreaks in ecological situations characterized by an absence of the usual peridomestic rodent plague reservoirs, the human flea was hypothesized as the vector in human-to-human transmission chains. Likely cases of human-to-human transmission by *P. irritans* have been documented in Ecuador, Peru, and Brazil [25, 33, 55]. However, because of the extremely high numbers of *P. irritans* common to human habitations in the Andean region, Macchiavello (1980) was of the opinion that if this flea was a significant vector, there would be many more plague outbreaks in that region (cited in [25]). Girard (1943) came to the same conclusion regarding the abundance and the potential role of *P. irritans* in Madagascar [42].

## Conclusions

Our results substantiate the reputation of *P. irritans* as a poor and unreliable vector of *Y. pestis*, particularly after feeding on bacteremic human blood. This is not primarily due to an inability of *Y. pestis* to stably infect *P. irritans*, but rather due to low transmission efficiency by both the early-phase and proventricular blockage mechanisms. Along with the considerations raised by others [36, 42, 53, 56, 61], these results cast doubt on the importance of *P. irritans* in driving human plague epidemics unless the flea burden were extremely high throughout a human population. Because of the difficulties in adapting *P. irritans* collected from non-human hosts to laboratory conditions used for vector competence studies, however, we recognize that further work is needed. Of particular importance will be the study of human-adapted *P. irritans* strains collected from human habitations in plague-endemic areas, and utilizing the host blood sources they are adapted to. Furthermore, much crucial information regarding the vectorial capacity and biogeography of this flea is still missing. To better understand its potential epidemiological importance, future studies should also address the temporal and geographical distribution and abundance of *P. irritans* in relation to human plague.

## Supplementary Information

**Additional file 1: Table S1.** Summary of the experiments.

**Additional file 2: Table S2.** Calculation of the blood meal volume of female *Pulex irritans* fleas.

## Data Availability

The datasets used and/or analysed during the current study are available from the corresponding author on reasonable request.
