# Supplementary material for: Poor vector competence of the human flea, Pulex irritans, to transmit Yersinia pestis
Source: Parasit Vectors. 2021 Jun 10;14:317. doi: 10.1186/s13071-021-04805-3 (PMC8194109; doi:10.1186/s13071-021-04805-3)
Supplement: Supplementary file 1 — Additional file 1: Table S1. Summary of the experiments. [file 13071_2021_4805_MOESM1_ESM.docx]

| **Table S1.** Summary of the experiments | | | | | | |
| --- | --- | --- | --- | --- | --- | --- |
| Figure | Flea source | Pre-infection maintenance blood meals^1^ | *Y. pestis* CFU/ml in infectious blood meal | Post-infection maintenance  blood meals^2^ | Length of the exp (days)^3^ | Flea no. (day 0) |
| Group I: Fleas infected using human blood and fed sterile human blood daily starting from day 2 after infection | | | | | | |
| 1A | Owl | 0, 1 (human) | 1.9 x 10^8^ | **1**, 2, **3**, 4, **5**, 6, **7** | 7 | 19 |
| 1B | Owl | None | 1.9 x 10^8^ | **1**, 2, **3**, 4, **5** | 5 | 16 |
|  |  |  |  |  |  |  |
| 1C | Fox | 0, 1, 2 (rat);  3, 4, 6 (mouse);  7, 8, 9 (human) | 5.1 x 10^8^ | **2**, 3 ,4, **5**, 6, 7, 8, **9**, 10, 11, 12, **13**, 14, 15, **16** | 16 | 60 |
| 1D | Owl | 0 (human) | 1.4 x 10^9^ | 1, **2**, 3, **4**, 5, **6**, 7, **8**, 9, **10**, 11, **12**, 13, **14**, 15, **16**, 17, **18**, 19, **20** | 20 | 52 |
| Group II: Fleas infected using human blood and subsequently fed sterile human blood every two days | | | | | | |
| 2A | Fox | 0, 2, 4, 6 (rat) | 7.9 x 10^8^ | **2, 4, 6, 8, 10, 12, 14, 16, 18, 20, 22, 24, 26** | 26 | 119 |
| 2B | Owl | 0, 1 (human) | 1.0 x 10^9^ | **2**, 3, **4**, 5, **6** | 6 | 12 |
| Group III: Fleas infected using rat blood and fed sterile rat blood daily starting from day 2 after infection | | | | | | |
| 3 | Fox | 3, 4, 5, 6 (rat) | 7 x 10^8^ | **2**, 3, 4, 5, 6, 7, **8**, 9, 10, 11, 12, 13, 14, **15** | 15 | 92 |
| Group IV: Fleas infected using rat blood and subsequently fed sterile rat blood every two to three days | | | | | | |
| 4A | Owl | None | 4.0 x 10^8^ | **3, 6, 8, 10** | 10 | 54 |
| 4B | Owl | None | 6.4 x 10^8^ | **3, 6, 8, 10** | 10 | 57 |
| 4C | Fox | 0, 2, 4, 6 (rat) | 3.0 x 10^8^ | **3, 5, 7, 9, 11, 13** | 13 | 108 |
| 4D | Fox | None | 9.9 x 10^8^ | **2**, **4**, **6**, **8**, **10**, **12** | 12 | 111 |
| 4E | Fox | 0, 2, 4, 6 (human) | 5.7 x 10^9^ | **2, 4, 6, 8, 10, 12, 14, 16, 18, 20, 22, 24, 26** | 26 | 124 |
| ^1^Fleas were maintained (or not) on sterile blood prior to the infection. The numbers represent the days when the fleas were fed, with day 0 the day they arrived in the laboratory; maintenance blood source is indicated in parentheses.  ^2^The days after infection when fleas received sterile maintenance blood meals, with day 0 being the day of the infectious blood meal. Transmission trials were conducted on days indicated in **bold**. Human blood was used for Group I and II experiments; rat blood for Groups III and IV.  ^3^The number of days from the day of infection (day 0) until the experiment was ended. | | | | | | |
|  | | | | | | |
