# Supplementary material for: Poor vector competence of the human flea, Pulex irritans, to transmit Yersinia pestis
Source: Parasit Vectors. 2021 Jun 10;14:317. doi: 10.1186/s13071-021-04805-3 (PMC8194109; doi:10.1186/s13071-021-04805-3)
Supplement: Supplementary file 2 — Additional file 2: Table S2. Calculation of the blood meal volume of female Pulex irritans fleas. [file 13071_2021_4805_MOESM2_ESM.docx]

| **Table S2.** Calculation of the blood meal volume of female *Pulex irritans* fleas | | |
| --- | --- | --- |
|  | pre-feed | post-feed |
| Combined weight | 8.504 mg  (n = 17 females) | 6.728 mg  (n = 8 of 17 that fed) |
| Avg. weight per flea | 0.500 mg | 0.841 mg |
| Avg. weight of blood meal |  | 0.341 mg |
| Avg. volume of blood meal* | | 0.32 μl |
| * Avg. weight divided by the specific gravity of blood (1.06 g/ml) | | |
